# Evaluation of Problem-Based Learning implementation in a College of Medicine, Kingdom of Saudi Arabia: a cross sectional comparative study

**DOI:** 10.1186/s12909-022-03347-1

**Published:** 2022-04-23

**Authors:** Manahel A. Almulhem, Jwaher A. Almulhem

**Affiliations:** 1 Medical Education Department, College of Medicine, Imam Abdulrahman bin Faisal University, Dammam, Saudi Arabia; 2 Medical Education Department, College of Medicine, King Saud University, Riyadh, Saudi Arabia

**Keywords:** PBL, Problem-based learning, Perspectives, Medical students, Facilitators, Small group learning, Case scenario, Facilitator role

## Abstract

**Background:**

Problem-Based Learning (PBL) is an innovative student-centered learning method that has been implemented in numerous medical colleges worldwide. However, the newly adopting PBL institutes may face challenges during its implementation. This study aims to evaluate PBL implementation in the medical college of Imam Abdulrahman bin Faisal University (IAU) from the facilitators’ and students’ perspectives.

**Methods:**

This is a cross-sectional study using a quantitative self-administered online questionnaire. The questionnaire evaluated PBL implementation using the three scales: small group learning, problem case scenario, and facilitator role. A total of 52 facilitators and 1289 students (from second to sixth years) were invited to participate in the study at the end of the 2019–2020 academic year.

**Results:**

Forty-six facilitators (88.46%) and 324 students (25.13%) responded to the questionnaire. There was an overall positive evaluation of PBL implementation. However, the facilitators’ rating was significantly higher than the students’ rating across the three scales. Regarding the small group learning, a significant difference was found between types of facilitation status (*p* = 0.017) and between trained and not trained facilitators (*p* = 0.029). In respect to the problem case scenario, there was a significant difference based on the types of facilitation status (*p* = 0.017) and facilitating tutorials related to the facilitator’s specialty (*p* = 0.004). Regarding the facilitator role, a significant difference was found between the academic year they had facilitated (*p* = 0.032). Female students rated the three scales significantly higher than male students (*p* < 0.001). Students aged between 24 and 25 years old and sixth years students also rated the three scales significantly higher than other students (*P* < 0.05).

**Conclusions:**

The participants rated PBL implementation positively as measured by the three scales rating. However, specific concerns have been highlighted that are related to group dynamics, training before starting PBL, relevancy of the case scenarios, and the facilitator’s role in nominating group members and providing feedback.

**Supplementary Information:**

The online version contains supplementary material available at 10.1186/s12909-022-03347-1.

## Background

The conventional didactic Lecture-Based Learning (LBL) method is a much-used learning modality in the medical education field [1], specifically in the Kingdom of Saudi Arabia (KSA). Although LBL is an effective method for short-term learning [2], promoting lifelong learning skills such as problem-solving has become a key goal of educators in the twenty-first century worldwide [3]. As a result, Saudi medical colleges have moved toward innovative learning methods such as Problem-Based Learning (PBL) that produce lifelong learners [4, 5].

PBL is a student-centred learning method that allows the students to determine their learning objectives from a problem case scenario [6]. In the first meeting, the students analyse the problem case scenario to produce questions. These questions are considered as learning objectives. Then, the self-study phase start when the students research the learning objectives independently or in a group then re-join for a second meeting to discuss and refine their researched information [7] where the instructor acts as a facilitator of the learning process [2]. Therefore, PBL resolved the issue of teaching a large amount of knowledge that previously was taught passively and was unrelated to clinical practice [8]. Accordingly, PBL improves the students’ generic skills such as critical thinking, problem-solving, communication, collaboration [9], and self-directed learning (SDL) skills [10].

Despite the widespread use of PBL, its implementation is still challenging [11]. Moallem (2019) argued that evaluating only the outcomes of PBL is insufficient to assess its success. Rather, the success of PBL is also affected by the evaluation of its implementation. Several studies have evaluated PBL implementation from the facilitators’ perspectives in different medical colleges globally [12–15]. A recent study was conducted in 16 universities in the Kingdom of Saudi Arabia (KSA) to obtain an overall facilitators’ evaluation of PBL implementation. Al-Shehri et al. (2018) found that most of the facilitators rated themselves positively during the sessions. They reported that they were skilled at introducing the case scenarios (67.5%), directing the discussion toward the learning objectives (76.8%), stimulating the students to think critically (71.5%), providing information only when necessary (65.6%), creating a competitive environment where all students were encouraged to participate in discussions (76.8%) and taking notes to provide feedback for the individual student and the whole group (68.3%). They concluded that PBL was implemented moderately (50%) across the universities in the KSA [12].

Likewise, several studies concentrated on PBL implementation from the students’ perspectives [16–18]. A study conducted in King Saud bin Abdulaziz University for Health Sciences reported that most of the students stated that the group members were cooperative, the discussions were organized, the role of each member was defined, and they accepted the feedback comments from each other. Moreover, 67% stated that the problem case scenario was a trigger for the group discussion [19].

Considering the perspectives of both the students and facilitators is equally important to improve the educational experience for both groups. Yet, the literature is lacking comparative studies between the facilitators’ and medical student’s perspectives [14]. Previous studies concentrated on facilitators’ perspectives [12–15] or students’ perspectives [16–19]. Furthermore, the findings of these studies were variable since each institute has its own context and experience, which is an important factor in the context of educational intervention [20]. Therefore, this study intends to improve PBL implementation at Imam Abdulrahman bin Faisal University (IAU) to enhance the educational experience and increase the body of literature regarding PBL implementation in medical education. Accordingly, this study aims to compare the medical students’ and facilitators’ evaluations of PBL implementation at IAU.

### Research questions and hypothesis

1. What is medical students’ evaluation of PBL implementation in the faculty of medicine at IAU, KSA?
2. What is facilitators’ evaluation of PBL implementation in the faculty of medicine at IAU, KSA?
3. What is the difference between the medical students’ and facilitators’ evaluations of PBL implementation in the faculty of medicine at IAU, KSA?

In this paper, we hypothesized that the facilitators would rate PBL implementation significantly higher than the medical students.

## Methods

### Conceptual framework

The conceptual framework of this study was based on the agreed characteristics of PBL worldwide [21]. These universal characteristics include small-group learning, facilitator role, and triggering problem. Evaluating the process of PBL implementation is an important aspect to assure the quality of education. Moallem [22] argued that evaluating only the outcomes of PBL is insufficient to assess its success. Rather, the success of PBL is also affected by the evaluation of its implementation. Considering the perspectives of different stakeholders is important because it reflects the broader view of any experience. Therefore, this study compared the evaluations of the facilitators and students to enhance the educational experience for both.

### IAU PBL curriculum

IAU implemented PBL as an educational method for second and third-year medical students since 2014 [23]. The curriculum follows a curriculum originated in an Australian university that is contextualized to the Saudi settings [24]. The curriculum was modified based on the Saudi local context that included local health needs, commonality of diseases, and cultural and social issues. The learning process includes facilitated sessions, students’ presentations, and small group discussions supported by clinical teaching [25] which makes it a hybrid PBL. The hybrid model incorporates PBL tutorials with conventional teaching methods such as lectures [26]. Training sessions take place at the beginning of the students’ second year and before tutors’ facilitation. The training sessions cover a wide range of topics such as what is PBL, how to apply PBL and its assessment methods. The material is presented in several forms including lectures, workshops, handbooks and observed sessions. The assessment of the training sessions is through post-session feedback and surveys. The facilitators were recruited by their department. Each department was responsible for nominating the facilitators based on the availability of the facilitators’ schedule and academic load.

Each tutorial consists of two sessions where a small group of approximately 14 students is provided with a real patient case scenario that integrates the material learned during the week. The students rotate through different roles in each tutorial which includes a leader, a scribe, and members. The group works both individually and collaboratively to identify their learning objectives. The problem case scenarios and learning objectives are similar to the Australian University cases, but they are contextualized to the Saudi settings [25].

At IAU, each new PBL facilitator attends a training session before facilitation. Also, the new facilitator must observe two PBL sessions with an experienced facilitator at IAU. As the initial group of facilitators was not able to observe an experienced facilitator at IAU, they were trained by the Australian University’s training experts.

### Study design and participants

This was a cross-sectional study conducted from June 2020 to July 2020. The study population included two groups of participants: the PBL facilitators and medical students at the IAU medical college. The first group comprised the current and the previous facilitators, a total population of 52 facilitators from different departments in the medical college. The second group included all the medical students, a total of 1289 students. The participants were identified through facilitators and students lists from the IAU medical education department.

### Inclusion criteria

All the current and previous facilitators who facilitated the second- and third-year PBL tutorials from male and female separate campuses were invited to participate in the study. The study included both the current and previous facilitators to collect a comprehensive evaluation from the start time when PBL implemented. All the students who completed the second to the sixth year of medical college were eligible to participate. The students from all the years were included because they had either a current or a previous experience of PBL. All of that was considered to compare PBL implementation at the beginning with it current status which helped in following the progress of PBL implementation.

### Exclusion criteria

The previous facilitators with no contact information and the facilitators who left the university permanently were excluded from the sample because they were inaccessible.

### Questionnaire development, piloting, and distribution

A self-developed questionnaire (Additional file 2: Appendix 1) was developed after reviewing multiple studies that concentrated on PBL evaluation [14, 27]. It consisted of four sections: the participant information sheet, informed consent, demographic data and evaluation of PBL implementation.

The facilitators’ demographic data included gender, age, department, educational background, academic background, academic status (job title) and facilitation status. Whereas the students provided demographic data relating to their gender, age, and academic level. The evaluation section of the questionnaire consisted of 29 items for both groups. The items were developed based on previous studies [12, 14, 28]. This section was divided into three main scales that represented the universally agreed characteristics of PBL, namely small group learning, problem case scenario, and facilitator role. A five-point scale (Never = 1, Rarely = 2, Sometimes = 3, Often = 4, and Always = 5) was used.

The questionnaire was reviewed by a PBL expert to ensure content validity. Also, it was piloted with three medical students in order to assess the layout, options, clarity, and understanding of the questionnaire instructions and items. The pilot study showed acceptance from the participants and provided feedback that was considered for questionnaire modification and development which included few wording and clarity issues.

Cochran formula was used to calculate the required sample size [29]. Based on the equation, the required sample size for the facilitators’ group was 46 participants while it was 297 participants for the students’ group.

The questionnaire was developed by Bristol Online Survey (BOS), which provided designing, distribution, and analysis services. A subscription was purchased by the University of Dundee and offered free of charge. To recruit the participants, a convenience sampling technique was used. All the facilitators and students who met the inclusion criteria were approached by the IAU Deanship of Scientific Research through email with an invitation that contained the study’s objective and a hyperlink that led to the questionnaire web page.

Accordingly, surveys were randomly distributed among the study population which included 1289 medical students and 52 facilitators. The target was to collect at least the required sample which were 297 students’ and 46 facilitators’ surveys. To increase response rate, the participants were reminded multiple times through different communication methods. The facilitators were approached through other messaging technologies such as Text messages, WhatsApp, and the university email.

### Ethical considerations

Ethical permission was obtained from the IAU Institutional Review Board (IRB) committee (IRB-PGS-2020-01-125) and the University of Dundee Research Ethics Committee (SMED REC Number 20/39). The information sheet included the purpose of the study, participants’ right to withdraw, ensuring confidentiality and anonymity, and information on data storage. An informed consent that confirmed their voluntary participation in the study was also included in the beginning of the questionnaire. The participants were informed that any identifiable data would not be published. The data will be stored in a securely password-protected database for a period of 5 years after completion of the study.

### Data analysis

The collected data was exported from BOS to Excel sheet then analysed by the Statistical Package for Social Sciences (SPSS) version 23.0. The data was analysed by descriptive statistics (percentages and means). In terms of frequency, always and often were considered as positive ratings while sometimes, rarely, and never were considered as negative ratings. Also, the t-test and One-way Analysis of Variance (ANOVA) were used to compare the evaluations of different groups. *P*-value ≤0.05 was considered a statistically significant association for both tests.

## Results

### Participants’ demographic characteristics

Forty-six out of 52 facilitators (24 female and 22 male) responded (a response rate of 88.46%). Three hundred and 24 out of 1289 medical students responded (a response rate of 25.13%).

The majority of the facilitators were between the ages of 45–54 (43.5%). The facilitators participated from several departments with Physiology (32.6%) being the most common. The majority graduated with a medical degree (95.6%), 58.7% were academic basic scientists (academic faculty from academic departments such as Physiology and Anatomy), and 41.3% were physicians. The majority of the academic basic scientists were assistant professors (66.7%) and the rest were lecturers (18.5%) and associate professors (14.8%). About two-thirds of the physicians (63.2%) were consultants and the remaining were specialists (36.8%). Most of the facilitator participants were current PBL facilitators (73.9%) while the rest were ex-facilitators (26.1%). About a third had 3–4 years of facilitation experience and about another third had 5–6 years of experience (See Supplementary Table 1, Additional file 1).

Regarding PBL facilitation characteristics, almost half of the facilitators facilitated only the second-year medical students’ tutorials (47.8%). About a quarter facilitated only third-year tutorials (28.2%) and the remainder facilitated both the second and third-year tutorials (23.9%). Most of the facilitators facilitated the PBL tutorials related to their specialty (69.5%). The majority of the academic basic scientists (81.5%) and about half of the physicians (52.6%) facilitated tutorials related to their specialty. The majority of the facilitators attended a training session (84.7%) and observed a PBL tutorial before facilitation (82.6%). The breakdown was the same for academic basic scientists and physicians (See Supplementary Table 1, Additional file 1).

Out of the 324 medical students, 221 were female (68.2%). The majority were between the ages of 20–21 (36.7%) and 22–23 years old (39.8%). Regarding their academic year, the students were evenly distributed between years (See Supplementary Table 1, Additional file 1).

### The facilitators' evaluation of PBL implementation

The facilitators’ evaluations of PBL implementation were uniformly positive across the three main scales (small group learning, problem case scenario, and facilitator role). Specifically, the overall means of each scale were more than 4.3 and the mean of each item was more than 4. The facilitator role scale was rated the highest and the problem case scenario scale the lowest, based on the mean (Tables 1 and 2).

**Table 1:** The facilitators’ rating of the small group learning and problem case scenario

	Item	No. (%)					Mean (SD)
		1 (Never)	2 (Rarely)	3 (Sometimes)	4 (Often)	5 (Always)	
**Small group learning**							
1	There is proper students’ training before starting the PBL.	2 (4.3)	1 (2.2)	8 (17.4)	9 (19.6)	26 (56.5)	4.22 (1.08)
2	The students themselves read the problem case scenario that is given by the facilitator.	0 (0)	0 (0)	1 (2.2)	3 (6.5)	42 (91.3)	4.89 (0.37)
3	The group leader engages all the students in the discussion equally.	0 (0)	0 (0)	7 (15.2)	27 (58.7)	12 (26.1)	4.11 (0.63)
4	The students have a chance to talk more than the facilitator.	0 (0)	1 (2.2)	2 (4.3)	11 (23.9)	32 (69.6)	4.61 (0.67)
5	The students express their point of view by hand-raising (without interrupting other students’ points of view).	1 (2.2)	0 (0)	6 (13)	28 (60.9)	11 (23.9)	4.04 (0.75)
6	The scribe writes the main point of the discussion.	0 (0)	0 (0)	1 (2.2)	8 (17.4)	37 (80.4)	4.78 (0.46)
7	The students themselves identify the learning objectives.	0 (0)	1 (2.2)	3 (6.5)	23 (50)	19 (41.3)	4.3 (0.69)
8	A student summarizes the case scenario at the beginning of the second part of the tutorial (second session).	2 (4.3)	3 (6.5)	5 (10.9)	9 (19.6)	27 (58.7)	4.22 (1.14)
9	The students do the presentation appropriately for the second part of the tutorial (second session).	0 (0)	0 (0)	3 (6.5)	14 (30.4)	29 (63)	4.57 (0.61)
10	A student summarizes the case scenario at the end of the second part of the tutorial (second session) “case integration”.	0 (0)	0 (0)	5 (10.9)	13 (28.3)	28 (60.9)	4.5 (0.68)
11	Learning resources (e.g. library, computers and IT support) are provided to support the learning process.	0 (0)	0 (0)	6 (13)	10 (21.7)	30 (65.2)	4.52 (0.71)
**Overall mean**							**4.43**
**Problem case scenario**							
1	PBL case scenarios are clearly written.	0 (0)	0 (0)	1 (2.2)	18 (39.1)	27 (58.7)	4.57 (0.54)
2	PBL case scenarios have an appropriate trigger.	0 (0)	0 (0)	4 (8.7)	18 (39.1)	24 (52.2)	4.43 (0.65)
3	PBL case scenarios generate a range of differential diagnoses (in the first session).	0 (0)	1 (2.2)	4 (8.7)	16 (34.8)	25 (54.3)	4.41 (0.74)
4	PBL case scenarios have an appropriate level of difficulty.	0 (0)	0 (0)	5 (10.9)	20 (43.5)	21 (45.7)	4.35 (0.67)
5	PBL case scenarios are related (contextualized) to local settings	0 (0)	3 (6.5)	6 (13)	18 (39.1)	19 (41.3)	4.15 (0.88)
**Overall mean**							**4.38**

**Table 2:** The facilitators’ rating of their role

	Item	No. (%)					Mean (SD)
		1 (Never)	2 (Rarely)	3 (Sometimes)	4 (Often)	5 (Always)	
1	The facilitator sets the ground rules.	0 (0)	0 (0)	3 (6.5)	10 (21.7)	33 (71.7)	4.65 (0.6)
2	The facilitator nominates the scriber for every new case scenario (on-turn).	4 (8.7)	3 (6.5)	4 (8.7)	7 (15.2)	28 (60.9)	4.13 (1.31)
3	The facilitator nominates the group leader for every new case scenario (on-turn).	4 (8.7)	2 (4.3)	4 (8.7)	5 (10.9)	31 (67.4)	4.24 (1.29)
4	The facilitator stimulates the students’ critical thinking by asking questions.	0 (0)	1 (2.2)	0 (0)	11 (23.9)	34 (73.9)	4.7 (0.58)
5	The facilitator stimulates the students to apply knowledge to the discussed problem.	0 (0)	1 (2.2)	0 (0)	11 (23.9)	34 (73.9)	4.7 (0.58)
6	The facilitator directs the students toward the learning objectives (gives hints for the uncovered objectives).	1 (2.2)	2 (4.3)	3 (6.5)	11 (23.9)	29 (63)	4.41 (0.95)
7	The facilitator ensures all students’ participation.	0 (0)	1 (2.2)	0 (0)	10 (21.7)	35 (76.1)	4.72 (0.58)
8	The facilitator takes notes of the students’ performance in the tutorial.	0 (0)	1 (2.2)	1 (2.2)	11 (23.9)	33 (71.7)	4.65 (0.63)
9	The facilitator marks the students for their participation in the tutorial.	1 (2.2)	1 (2.2)	1 (2.2)	1 (2.2)	42 (91.3)	4.78 (0.78)
10	The facilitator provides feedback on individual students’ performance after the tutorial if needed.	1 (2.2)	1 (2.2)	4 (8.7)	12 (26.1)	28 (60.9)	4.41 (0.9)
11	The facilitator provides individual feedback privately if needed.	0 (0)	1 (2.2)	5 (10.9)	12 (26.1)	28 (60.9)	4.46 (0.77)
12	The facilitator has time management skills.	0 (0)	1 (2.2)	1 (2.2)	12 (26.1)	32 (69.6)	4.63 (0.64)
13	The facilitator fulfills her or his role as a facilitator.	0 (0)	1 (2.2)	1 (2.2)	9 (19.6)	35 (76.1)	4.7 (0.62)
**Overall mean**							**4.55**

Regarding the small group learning, the most highly rated statements related to reading the case scenario (4.89) and the scribe role (4.78). The majority of the facilitators stated that the students always read the problem case scenario (91.3%) and the scribe always wrote the main point of the discussion (80.4%). On the other hand, the leader role (4.11) and respect between the group members (4.04) were rated the least. Less than a third of the facilitators agreed that the group leader always engaged all the students in the discussion equally (26.1%) and the students always expressed their point of view by hand-raising (without interrupting other students’ points of view) (23.9%) (Table 1).

In respect to the problem case scenario, the clarity of the cases was highly rated (4.57), while the relation of the cases to the local setting was the least rated (4.15) (Table 1).

The facilitators rated their overall role as the highest of the three scales (4.55). Marking the students during the tutorial (4.78) and ensuring all students’ participation (4.72) were the highest-rated items. The majority of the facilitators stated that they always marked the students for their participation in the tutorial (91.3%) and ensured all students’ participation (76.1%). On the other hand, nominating the group members for the scribe (4.13) and leader (4.24) roles were the lowest rated. Around two-thirds of the facilitators stated that they nominated the scriber (60.9%) and group leader (67.4%) for every new case scenario (on-turn) (Table 2).

### The students’ evaluation of PBL implementation

Generally, the students rated the PBL implementation positively; however, they rated each scale lower than the facilitators. The overall mean of each scale was 4.04 for the problem case scenario, 3.98 for small group learning, and 3.97 for the facilitator role (Tables 3 and 4).

**Table 3:** The students’ rating of the small group learning and problem case scenario

	Item	No. (%)					Mean (SD)
		1 (Never)	2 (Rarely)	3 (Sometimes)	4 (Often)	5 (Always)	
**Small group learning**							
1	There is proper students’ training before starting the PBL.	49 (15.1)	66 (20.4)	89 (27.5)	67 (20.7)	53 (16.4)	3.03 (1.29)
2	The students themselves read the problem case scenario that is given by the facilitator.	6 (1.9)	9 (2.8)	20 (6.2)	45 (13.9)	244 (75.3)	4.58 (0.87)
3	The group leader engages all the students in the discussion equally.	8 (2.5)	32 (9.9)	76 (23.5)	101 (31.2)	107 (33)	3.82 (1.07)
4	The students have a chance to talk more than the facilitator.	5 (1.5)	9 (2.8)	58 (17.9)	111 (34.3)	141 (43.5)	4.15 (0.92)
5	The students express their point of view by hand-raising (without interrupting other students’ points of view).	13 (4)	34 (10.5)	74 (22.8)	115 (35.5)	88 (27.2)	3.71 (1.09)
6	The scribe writes the main point of the discussion.	5 (1.5)	6 (1.9)	40 (12.3)	68 (21)	205 (63.3)	4.43 (0.89)
7	The students themselves identify the learning objectives.	2 (0.6)	14 (4.3)	46 (14.2)	115 (35.5)	147 (45.4)	4.21 (0.88)
8	A student summarizes the case scenario at the beginning of the second part of the tutorial (second session).	19 (5.9)	33 (10.2)	70 (21.6)	81 (25)	121 (37.3)	3.78 (1.21)
9	The students do the presentation appropriately for the second part of the tutorial (second session).	3 (0.9)	13 (4)	31 (9.6)	84 (25.9)	193 (59.6)	4.39 (0.88)
10	A student summarizes the case scenario at the end of the second part of the tutorial (second session) “case integration”.	24 (7.4)	35 (10.8)	53 (16.4)	76 (23.5)	136 (42)	3.82 (1.28)
11	Learning resources (e.g. library, computers, and IT support) are provided to support the learning process.	17 (5.2)	29 (9)	59 (18.2)	87 (26.9)	132 (40.7)	3.89 (1.19)
**Overall mean**							**3.98**
**Problem case scenario**							
1	PBL case scenarios are clearly written.	8 (2.5)	10 (3.1)	48 (14.8)	120 (37)	138 (42.6)	4.14 (0.95)
2	PBL case scenarios have an appropriate trigger.	8 (2.5)	14 (4.3)	54 (16.7)	129 (39.8)	119 (36.7)	4.04 (0.96)
3	PBL case scenarios generate a range of differential diagnoses (in the first session).	6 (1.9)	12 (3.7)	63 (19.4)	111 (34.3)	132 (40.7)	4.08 (0.95)
4	PBL case scenarios have an appropriate level of difficulty.	6 (1.9)	11 (3.4)	68 (21)	132 (40.7)	107 (33)	4.0 (0.92)
5	PBL case scenarios are related (contextualized) to local settings	8 (2.5)	18 (5.6)	72 (22.2)	107 (33)	119 (36.7)	3.96 (1.02)
**Overall mean**							**4.04**

**Table 4:** The students’ rating of the facilitator role

	Item	No. (%)					Mean (SD)
		1 (Never)	2 (Rarely)	3 (Sometimes)	4 (Often)	5 (Always)	
1	The facilitator sets the ground rules.	8 (2.5)	6 (1.9)	39 (12)	66 (20.4)	205 (63.3)	4.4 (0.94)
2	The facilitator nominates the scriber for every new case scenario (on-turn).	15 (4.6)	23 (7.1)	55 (17)	67 (20.7)	164 (50.6)	4.06 (1.17)
3	The facilitator nominates the group leader for every new case scenario (on-turn).	17 (5.2)	27 (8.3)	39 (12)	63 (19.4)	178 (54.9)	4.1 (1.21)
4	The facilitator stimulates the students’ critical thinking by asking questions.	5 (1.5)	8 (2.5)	42 (13)	123 (38)	146 (45.1)	4.23 (0.88)
5	The facilitator stimulates the students to apply knowledge to the discussed problem.	4 (1.2)	11 (3.4)	44 (13.6)	117 (36.1)	148 (45.7)	4.22 (0.89)
6	The facilitator directs the students toward the learning objectives (gives hints for the uncovered objectives).	5 (1.5)	10 (3.1)	40 (12.3)	112 (34.6)	157 (48.5)	4.25 (0.9)
7	The facilitator ensures all students’ participation.	4 (1.2)	16 (4.9)	57 (17.6)	93 (28.7)	154 (47.5)	4.16 (0.97)
8	The facilitator takes notes of the students’ performance in the tutorial.	9 (2.8)	27 (8.3)	62 (19.1)	89 (27.5)	137 (42.3)	3.98 (1.09)
9	The facilitator marks the students for their participation in the tutorial.	7 (2.2)	17 (5.2)	49 (15.1)	89 (27.5)	162 (50)	4.18 (1.01)
10	The facilitator provides feedback on individual students’ performance after the tutorial if needed.	18 (5.6)	51 (15.7)	96 (29.6)	79 (24.4)	80 (24.7)	3.47 (1.18)
11	The facilitator provides individual feedback privately if needed.	61 (18.8)	54 (16.7)	77 (23.8)	65 (20.1)	67 (20.7)	3.07 (1.39)
12	The facilitator has time management skills.	17 (5.2)	44 (13.6)	94 (29)	98 (30.2)	71(21.9)	3.5 (1.13)
13	The facilitator fulfills her or his role as a facilitator.	8 (2.5)	9 (2.8)	54 (16.7)	126 (38.9)	127 (39.2)	4.1 (0.94)
**Overall mean**							**3.97**

With respect to the small group learning, reading the case scenario (4.58) and the scribe role (4.43) were the highest rated items. The majority of the students stated that they always read the problem case scenario (75.3%) and the scribe always wrote the main point of the discussion (63.3%). Training before PBL (3.03) and respect between the group members (3.71) were rated the lowest in terms of frequency. Less than a fifth stated that they always received proper training before starting the PBL (16.4%) and about a quarter expressed their point of view by hand-raising during the discussion (27.3%) (Table 3).

The students rated the problem case scenario scale higher than the other scales. The clarity of the cases was rated the highest (4.14) while the relation of the case to the local settings was rated the lowest (3.96) (Table 3).

The students rated the role of their facilitators scale the lowest. Nevertheless, setting ground rules (4.4) and directing the discussion (4.25) were rated highly. The majority stated that the facilitators always directed the students toward the learning objectives (63.3%) and around half of them agreed that they set the ground rules (48.5%). However, providing feedback afterward (3.47) and in private settings (3.07) were rated relatively low (Table 4).

### The facilitators’ characteristics and the evaluation scales

#### Small group learning scale

There were no statistically significant differences based on gender, educational background, academic background, whether the facilitators facilitated tutorials related to their specialty, and observed tutorial before facilitation. However, a significant difference was found between the types of facilitation status (*p = 0*.017)*.* Current facilitators evaluated the small group learning significantly higher than ex-facilitators with mean score 49.79 ± 3.641 vs. 45.83 ± 7.095. A similar significant difference was found between facilitators who attended a training session and did not attend (*p = 0*.029*)*. Facilitators who attended a training session before facilitation (49.44 ± 4.500) rated small group learning significantly higher than those who did not attend (45.00 ± 6.377) (Additional file 2: Appendix 2).

ANOVA test showed no significant differences between the age groups, departments, types of academic status, number of facilitation years, and academic year of facilitation (Additional file 2: Appendix 2).

#### Problem case scenario scale

There were no significant differences based on gender, educational background, academic background, attending a training session, and observing a PBL tutorial before facilitation. However, there was a significant difference between the types of facilitation status (*p = 0*.017*).* Current facilitators evaluated the problem case scenario scale significantly higher than the ex-facilitators with mean score 22.47 *±* 2.149 vs. 20.33 ± 3.525. Moreover, there was a significant difference regarding facilitating tutorials related to the facilitator’s specialty (*p = 0*.004*)*. Facilitators who facilitated tutorials related to their specialty *(*22.66 ± 2.548) evaluated the problem case scenario higher than those who facilitated tutorials not related to their specialty (20.21 ± 2.326) (Additional file 2: Appendix 3).

There were no significant differences between the different age groups, departments, types of academic status, number of facilitation years and the academic years they had facilitated (Additional file 2: Appendix 3).

#### Facilitator role scale

There were no significant differences based on gender, educational background, academic background, types of facilitation status whether the facilitators facilitated tutorials related to their specialty, attended a training session and observed a PBL tutorial before facilitation. Also, there were no significant differences between different age groups, departments, types of academic status, and number of facilitation years (Additional file 2: Appendix 4).

However, there was a significant difference between the academic year they had facilitated *F* (2, 43)= 3.727, *p* = 0.032. A Tukey post-hoc test was conducted for the different academic years of facilitation. It showed that a significant difference existed for the facilitators who facilitated both second- and third-year students (*p = 0*.039) (*M* = 62) (Additional file 2: Appendix 4).

### The students’ demographics and the evaluation scales

#### Small group learning

A statistically significant difference was found based on gender (*p = 0*.000). Female students evaluated the small group learning scale significantly higher with mean score of 42.56 *±* 5.354 vs 36.97 *±* 7.796). A similar significant difference was found based on age *F*(4,319) = 10.183, *p* = 0.000, and academic year the student has completed *F*(4,319) = 8.335, *p* = 0.000. Tukey post-hoc tests showed that a significant difference existed for the students aged between 22–23 and 24–25 years old as well as the fifth-year and sixth-year students (Additional file 2: Appendix 5).

#### Problem case scenario

Based on gender, there was a statically significant difference (*p = 0*.000). Female students evaluated the problem case scenario scale significantly higher based on the frequency scale with mean score of 20.81 *±* 3.407 vs18.96 *±* 4.542. Similarly, there was a significant difference based on age *F*(4,319) = 3.449, *p* = 0.009, and the academic year the student has completed *F*(4,319) = 2.521, *p* = 0.041. Tukey post-hoc tests showed that a significant difference existed for the students aged 24–25 and the sixth-year students (Additional file 2: Appendix 6).

#### Facilitator role

There was a statically significant difference based on gender (*p = 0*.000). Female students evaluated the facilitator role scale significantly higher with mean score of 53.42 *±* 7.526 vs. 48.07 *±* 11.049. A similarly significant difference was found based on the students’ age *F*(4,319) = 7.093, *p* = 0.000, and the academic year the student has completed *F*(4,319) = 4.652, *p* = 0.001. A statically significant difference existed for the students aged 24–25 and for the fifth-year and sixth-year students (Additional file 2: Appendix 7).

### Comparison between the facilitators’ and students’ evaluations

**Fig. 1:**
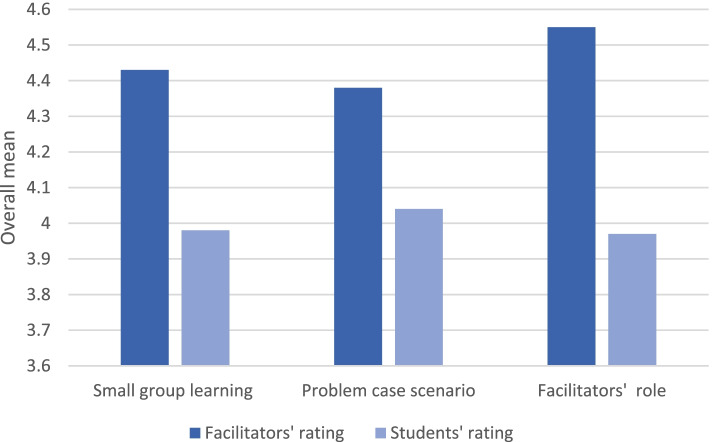
Comparison between the facilitators’ and students’ ratings of the three scales

A statistically significant difference was found on the three scales; small group learning (*p = 0*.000), problem case scenario scales (*p = 0*.005), and facilitator role (*p = 0*.000). The facilitators rated small group learning scale, problem case scenario scale, and facilitator role scale significantly higher than the students (48.76, ± 5.012 vs. 40.78 ± 6.746), *(*21.91 *±* 2.707 vs. 20.22 ± 3.894), (59.17 *±* 7.138 vs 51.72 ± 9.130) respectively (Fig. 1, Additional file 2: Appendix 8).

## Discussion

The aim of this study was to compare the medical students’ and facilitators’ evaluations of PBL implementation at IAU. This comparison enhances the body of the literature by providing background knowledge regarding PBL implementation. In addition, it assists the medical colleges that have not implemented PBL yet by providing them with more information to make an informed decision about their methods of curriculum evaluation. Despite the overall positive evaluation of PBL implementation in the medical college at IAU, there was a difference between the facilitators’ and students’ ratings. Therefore, the current study highlighted the importance of collecting different perspectives to evaluate PBL implementation. Collecting the perspectives of different stakeholders such as facilitators and students helps the institute to define their educational demands such as an effective feedback system, training sessions and fully equipped libraries. Defining such demands will help to meet these needs and enhance the educational experience for both.

Generally, the facilitators’ ratings were higher than the students’ ratings in multiple aspects. Further, it was shown that the current facilitators rated PBL significantly higher than other facilitators. This suggests that current facilitators are applying PBL concepts more frequently than the previous facilitators. Also, trained and experienced facilitators as well as facilitators who facilitated tutorials related to their specialty rated PBL significantly higher than other facilitators. This highlights the importance of training programme and assigning tutors to facilitate tutorials related to their specialty.

Regarding the students, female students rated PBL implementation significantly higher than male students. This suggests that teaching students on different campuses with different facilitators may influence the educational experience of the students at IAU. Also, older students and students in the advanced academic years rated PBL implementation significantly higher than other students. This indicates that the number of years that the students studied by PBL may be a factor that affected their ratings.

### Small group learning

In the present study, almost all the facilitators reported that the students always read the problem case scenario and the scribe always or often wrote the main discussion points, which were approved also by the students. This finding was opposed to another local study [14].

The present study also revealed that the facilitators and the students agreed on the lowest-rated items such as receiving proper training before the start of PBL, which was in agreement with other studies [27, 28]. This lack of proper students’ training may explain the lower students’ ratings of the three main scales when compared with the facilitators’ rating. Finding similar results in other local studies suggests that Saudi universities are still in need for more guidance to implement PBL properly and the first step starts with a training programme.

Another low rated item is the ability of expressing point of view by hand-raising and without interrupting other students. Such an attitude can affect the mutual respect between the group members which can produce an unfriendly learning environment. This may be attributed to the students’ teaching system before entering the university in the KSA and may affect the development of Saudi students’ communication skills. The learning system is mostly based on a teacher-centred method where the students are taught passively [30]. Such a passive learning attitude may lead to the underdevelopment of the student’s communication skills [31] such as choosing the right time to talk (respect for the turn).

### Problem case scenario

In the current study, both facilitators and students were agreed that the problem case scenarios were clear, which were inconsistent with another study that concerned the facilitator perspective [12]. The clarity of case scenarios indicated that the curriculum developers are aware of the students’ level of English proficiency. Clearly-written cases enhance the students’ achievement through positively influence on group discussion, identification of learning objectives, and self-study period [32]. On the other hand, more than a quarter of the students reported that the problem case scenarios were infrequently related to the local settings, as did a fifth of the facilitators. This is consistent with the results of a previous study which concentrated on the students’ perspective [19]. Providing students with real-world problems related to local settings will prepare them to recall and apply the same knowledge to real-world problems in the future [33, 34].

### Facilitator role

In the present study, facilitators and students responded positively regarding (ensuring the participation of all group members\students), which was similar to another study [12]. The interaction between the group members is based on sharing questions and knowledge as well as refining each other’s’ ideas and explanations [7]. Therefore, such participation enhances the students’ knowledge construction process. Additionally, the findings support the idea that the facilitators were capable of controlling the group dynamics, despite the presence of a considerable number of students who might likely interrupt each other, which is a major factor that influences the group interaction and enhance learning experience [35].

The majority of the facilitators stated that they either always or often stimulated the students’ critical thinking by asking questions and directed the students toward the learning objectives. The students also agreed with these findings, which was similar to another study [12]. Directing the discussion by asking challenging questions helps the students to think deeply and critically [36], explain their thoughts, and keeps them involved in the discussion [37]. Also, encouraging them to apply previous knowledge to the present case scenario will prepare them to apply their knowledge to real-world cases in the future.

On the other hand, more than half of the students stated that the facilitator infrequently or never provided feedback afterwards or in private settings. However, less than a sixth of the facilitators agreed that this was the case. The facilitators’ feedback is essential to monitor the students’ performance [38]. Therefore, such lack of feedback may influence the students’ learning process. They may not be able to identify their strengths and weaknesses during the discussion and self-study period. They also may lose the sense of progress that may lead to demotivation which in turn may hinder their learning process. Another study showed that the majority of Canadian students from five universities received individual and group feedbacks. However, the timing of the feedback was variable. The facilitators of the university that implemented PBL since the sixties provided immediate feedback while the other universities that introduced PBL in the last few years provided delayed feedback after one to several weeks [39]. This may explain the reason for the lack of feedback at IAU as it adopted PBL in 2014. In fact, insufficient teacher skills to provide feedback is a factor that influences feedback effectiveness [40]. Therefore, these skills may attribute to the students’ low rating of feedback item in this study.

In future, it is recommended to conduct a longitudinal study that will help to evaluate PBL implementation over a prolonged period of time [41]. Such studies will assist in following the progress of PBL implementation which will provide more valid data. In addition, a qualitative study is recommended to explore the facilitators’ and students’ experience in-depth and understand the factors that affected their ratings [42].

This study is subject to several limitations. The study was conducted at only one institute through using convenience sampling, which may have impact on external validity and generality of the findings. Another limitation which may have affect on generalizability of our finding is response rate. The students’ response rate was 25.13% despite sending multiple reminders and contacting them through various communication methods. This may attribute to the timing of data collection as the students are less likely to respond to questionnaires during vacation [43]. In addition, online questionnaires achieve lower response rates when compared with the paper-based type [20]. Furthermore, the survey was only validated through including expert review and small sample polit study. Moreover, factor analysis was not conducted due to small sample size and time constraints. Finally, the study may subject to recall bias because it included the previous facilitators and the students who had experienced PBL in previous academic years. However, they were targeted to compare their evaluation with the current facilitators’ and students’ evaluation in order to reach to a comprehensive evaluation of PBL implementation.

## Conclusion

The current study evaluated PBL implementation from the facilitators’ and students’ perspectives. The findings from this study showed that the curriculum developers should recognize the learning needs of the students and facilitators in order to improve the educational system. Although the response rate was low, the participants rated the PBL implementation positively as measured by frequency rating. However, the facilitators’ evaluation of the three main PBL characteristics was significantly higher than the students’ evaluation. This suggests that PBL is probably implemented well in the medical college at IAU from the facilitators’ point of view. It is recommended that providing proper training for both the facilitators and students would ensure better implementation of PBL in the medical colleges.

## Supplementary Information

**Additional file 1: Supplementary Table 1.** Distribution of the facilitators (*N* = 46) and students (*N* = 324) by demographic, professional and academic characteristics.

**Additional file 2: Appendix 1**: The web-based questionnaire. **Appendix 2**: The facilitators’ characteristics and the small group learning scale. **Appendix 3**: The facilitators’ characteristics and the problem case scenario scale. **Appendix 4**: The facilitators’ characteristics and the facilitator role scale. **Appendix 5**: The students’ characteristics and the small group learning scale. **Appendix 6**: The students’ characteristics and the problem case scenario scale. **Appendix 7**: The students’ characteristics and the facilitator role scale. **Appendix 8**: Comparison between the facilitators’ and students’ evaluations.

## Data Availability

The datasets generated and analysed during the current study are not publicly available to protect the confidentiality of the participants’ data but are available from the corresponding author on reasonable request.

## Abbreviations

PBL: Problem-Based Learning
IAU: Imam Abdulrahman bin Faisal University
SDL: Self-Directed Learning
KSA: Kingdom of Saudi Arabia
