# Supplementary material for: Evaluation of Problem-Based Learning implementation in a College of Medicine, Kingdom of Saudi Arabia: a cross sectional comparative study
Source: BMC Med Educ. 2022 Apr 23;22:311. doi: 10.1186/s12909-022-03347-1 (PMC9035263; doi:10.1186/s12909-022-03347-1)
Supplement: Supplementary file 1 — Additional file 1: Supplementary Table 1. Distribution of the facilitators (N = 46) and students (N = 324) by demographic, professional and academic characteristics. [file 12909_2022_3347_MOESM1_ESM.docx]

Table 1: Distribution of the facilitators (N=46) and students (N=324) by demographic, professional and academic characteristics.

| **Item** | | **No.** | **Percentage (%)** |
| --- | --- | --- | --- |
| **Facilitators’ characteristics** | | | |
| **Gender** | |  |  |
| Male | | 22 | 47.8 |
| Female | | 24 | 52.2 |
| **Age** | |  |  |
| 25-34 years old | | 8 | 17.4 |
| 35-44 years old | | 12 | 26.1 |
| 45-54 years old | | 18 | 39.1 |
| 55-64 years old | | 7 | 15.2 |
| 65 years old or more | | 1 | 2.2 |
| **Department** | |  |  |
| Anatomy | | 10 | 21.7 |
| Biochemistry | | 2 | 4.3 |
| FAMCO (Family and Community Medicine) | | 7 | 15.2 |
| Microbiology | | 3 | 6.5 |
| Pathology | | 3 | 6.5 |
| Physiology | | 15 | 32.6 |
| VDAA (Vice Dean of Academic Affairs) | | 4 | 8.7 |
| Other | | 2 | 4.3 |
| **Educational background** | |  |  |
| Medical background  (Graduated with MBBS) | | 44 | 95.70 |
| Non-medical background | | 2 | 4.30 |
| **Academic background** | |  |  |
| Physician | | 19 | 41.3 |
| Academic basic scientist | | 27 | 58.7 |
| **Academic status (job title)** | |  |  |
| Academic basic scientist | Demonstrator  (Teaching assistant) | 0 | 0 |
|  | Lecturer | 5 | 18.50 |
|  | Assistant professor | 18 | 66.70 |
|  | Associate Professor | 4 | 14.80 |
|  | Professor | 0 | 0 |
| Physicians | Residents | 0 | 0 |
|  | Specialists | 7 | 36.80 |
|  | Consultants | 12 | 63.20 |
| **Facilitation status** | |  |  |
| Current facilitator | | 34 | 73.91 |
| Previous facilitator | | 12 | 26.09 |
| **Number of facilitation years** | |  |  |
| 1-2 years | | 12 | 26.1 |
| 3-4 years | | 15 | 32.6 |
| 5-6 years | | 15 | 32.6 |
| > 6 years | | 4 | 8.7 |
| **The academic year the facilitator had facilitated** | |  |  |
| Second-year | | 22 | 47.8 |
| Third year | | 13 | 28.3 |
| Both second and third year | | 11 | 23.9 |
| **The facilitators’ experience of facilitating tutorials related to their specialty** | |  |  |
| Related to the facilitator's specialty | | 32 | 69.6 |
| Related to the facilitator's specialty | | 14 | 30.4 |
| **Attending training session before facilitation** | |  |  |
| Attended a training session before facilitation | | 39 | 84.8 |
| Did not attend a training session before facilitation | | 7 | 15.2 |
| **Observing training session before facilitation** | |  |  |
| Observed a PBL tutorial before facilitation | | 38 | 82.6 |
| Did not observe a PBL tutorial before facilitation | | 8 | 17.4 |
| **Students’ characteristics** | | | |
| **Gender** | |  |  |
| Male | | 103 | 31.8 |
| Female | | 221 | 68.2 |
| **Age** | |  |  |
| 18-19 years old | | 27 | 8.3 |
| 20-21 years old | | 119 | 36.7 |
| 22-23 years old | | 129 | 39.8 |
| 24-25 years old | | 46 | 14.2 |
| 26-27 years old | | 2 | 0.6 |
| > 27 years old | | 1 | 0.3 |
| **The academic year the student had completed** | |  |  |
| Second-year | | 62 | 19.1 |
| Third-year | | 52 | 16 |
| Fourth-year | | 72 | 22.2 |
| Fifth-year | | 75 | 23.1 |
| Sixth year | | 63 | 19.4 |
