# Supplementary material for: Evaluation of Problem-Based Learning implementation in a College of Medicine, Kingdom of Saudi Arabia: a cross sectional comparative study
Source: BMC Med Educ. 2022 Apr 23;22:311. doi: 10.1186/s12909-022-03347-1 (PMC9035263; doi:10.1186/s12909-022-03347-1)
Supplement: Supplementary file 2 — Additional file 2: Appendix 1: The web-based questionnaire. Appendix 2: The facilitators’ characteristics and the small group learning scale. Appendix 3: The facilitators’ characteristics and the problem case scenario scale. Appendix 4: The facilitators’ characteristics and the facilitator role scale. Appendix 5: The students’ characteristics and the small group learning scale. Appendix 6: The students’ characteristics and the problem case scenario scale. Appendix 7: The students’ characteristics and the facilitator role scale. Appendix 8: Comparison between the facilitators’ and students’ evaluations. [file 12909_2022_3347_MOESM2_ESM.docx]

# Appendix

## Appendix 1: The web-based questionnaire


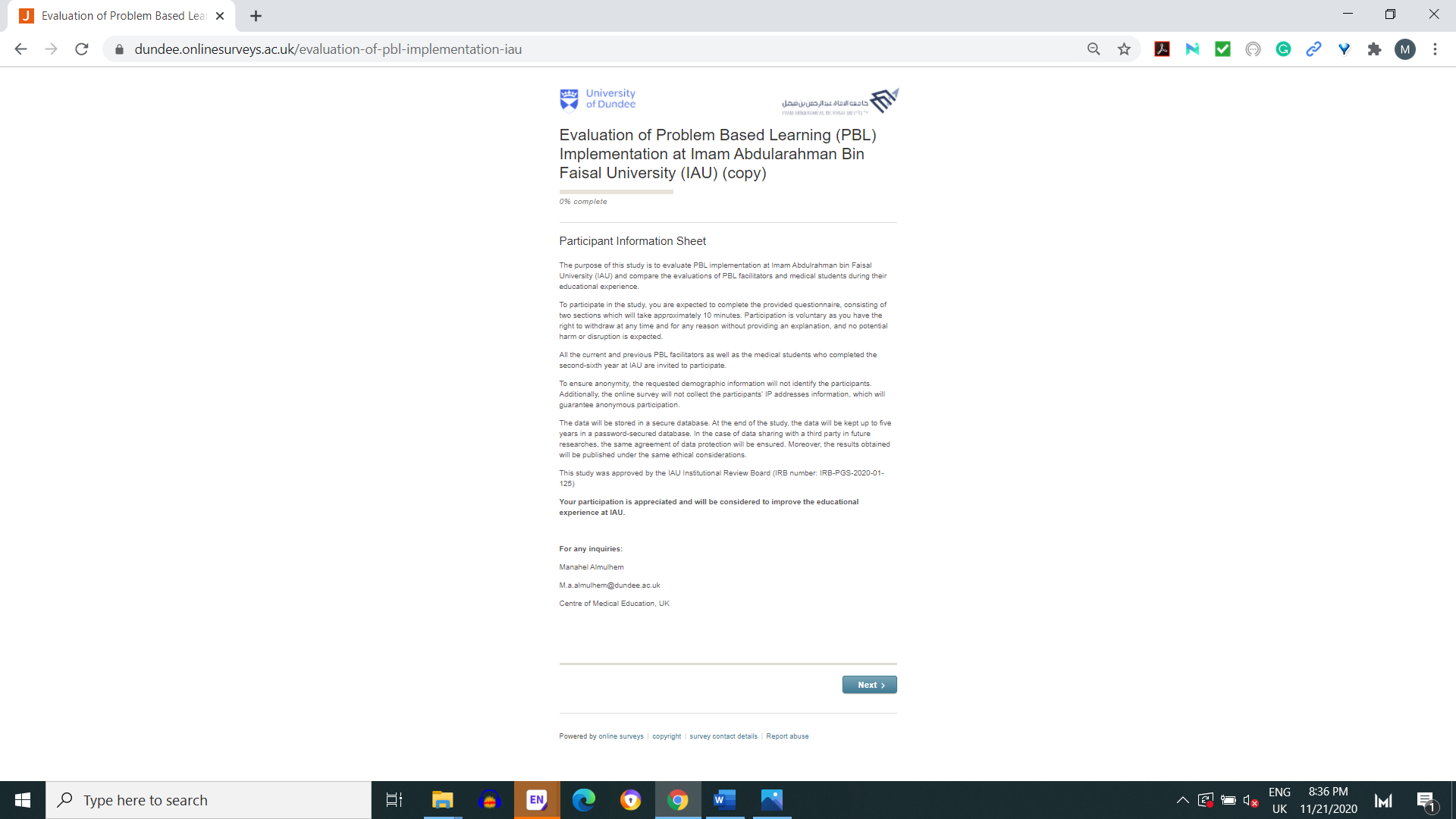


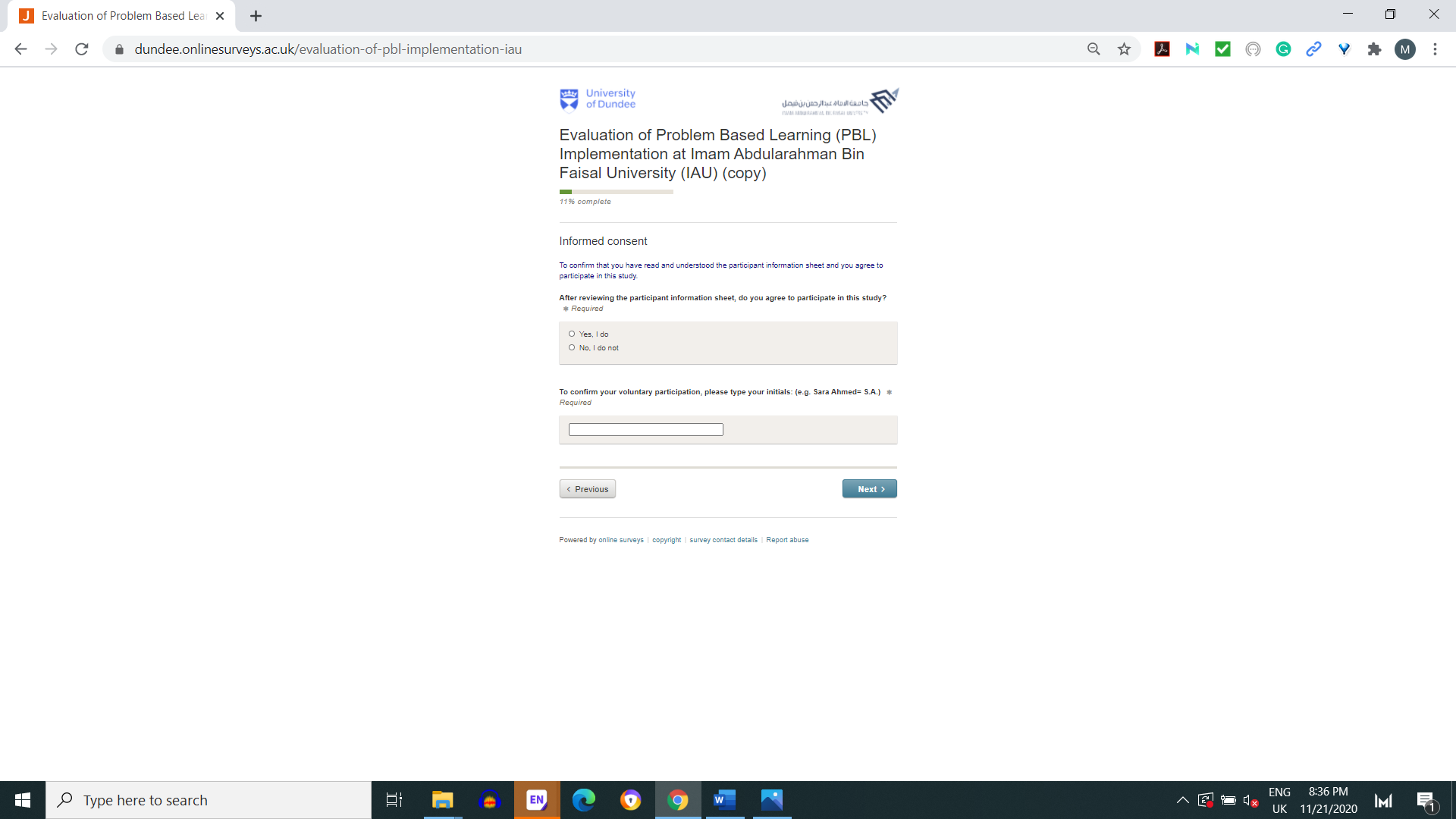


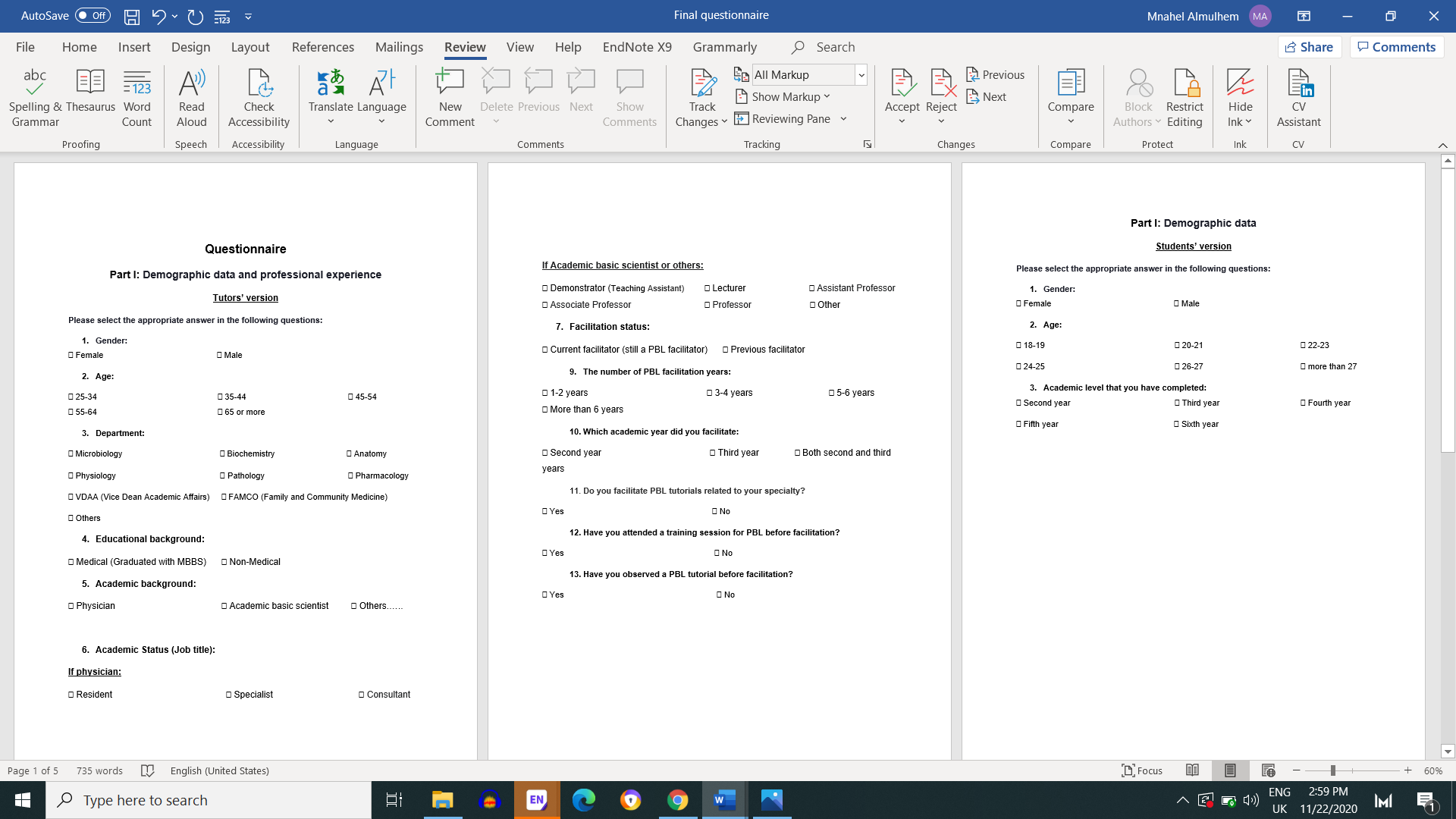


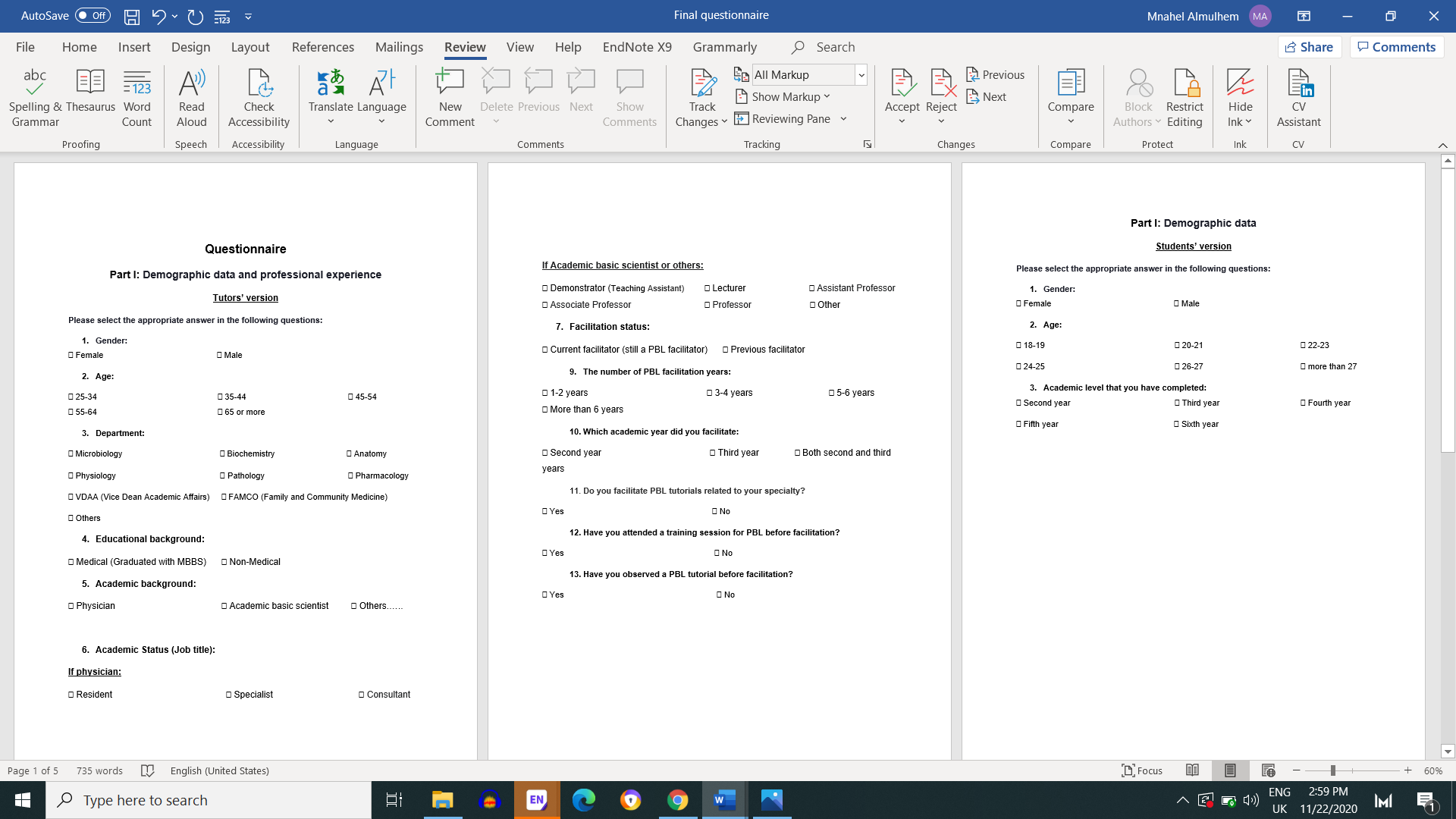


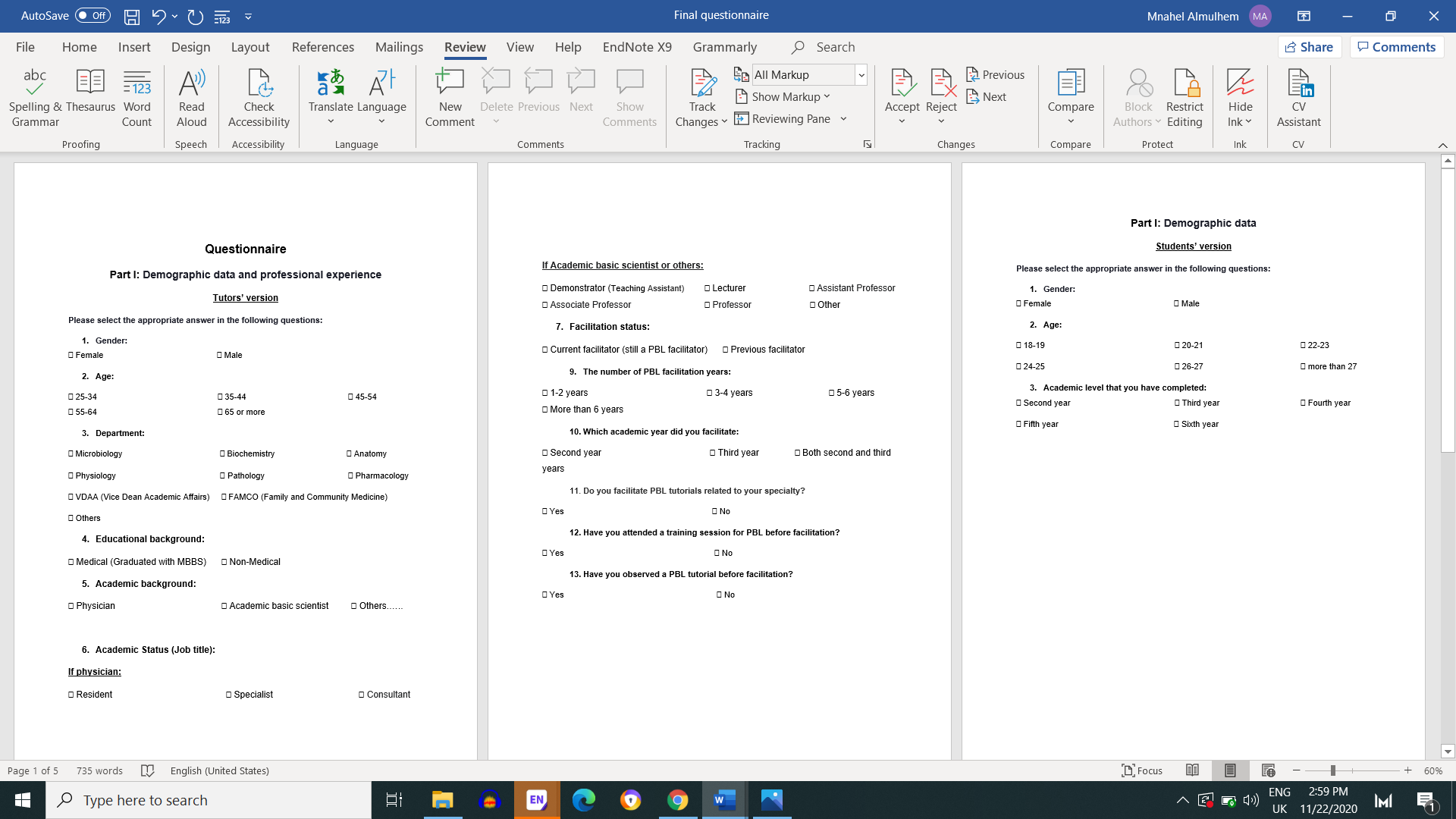


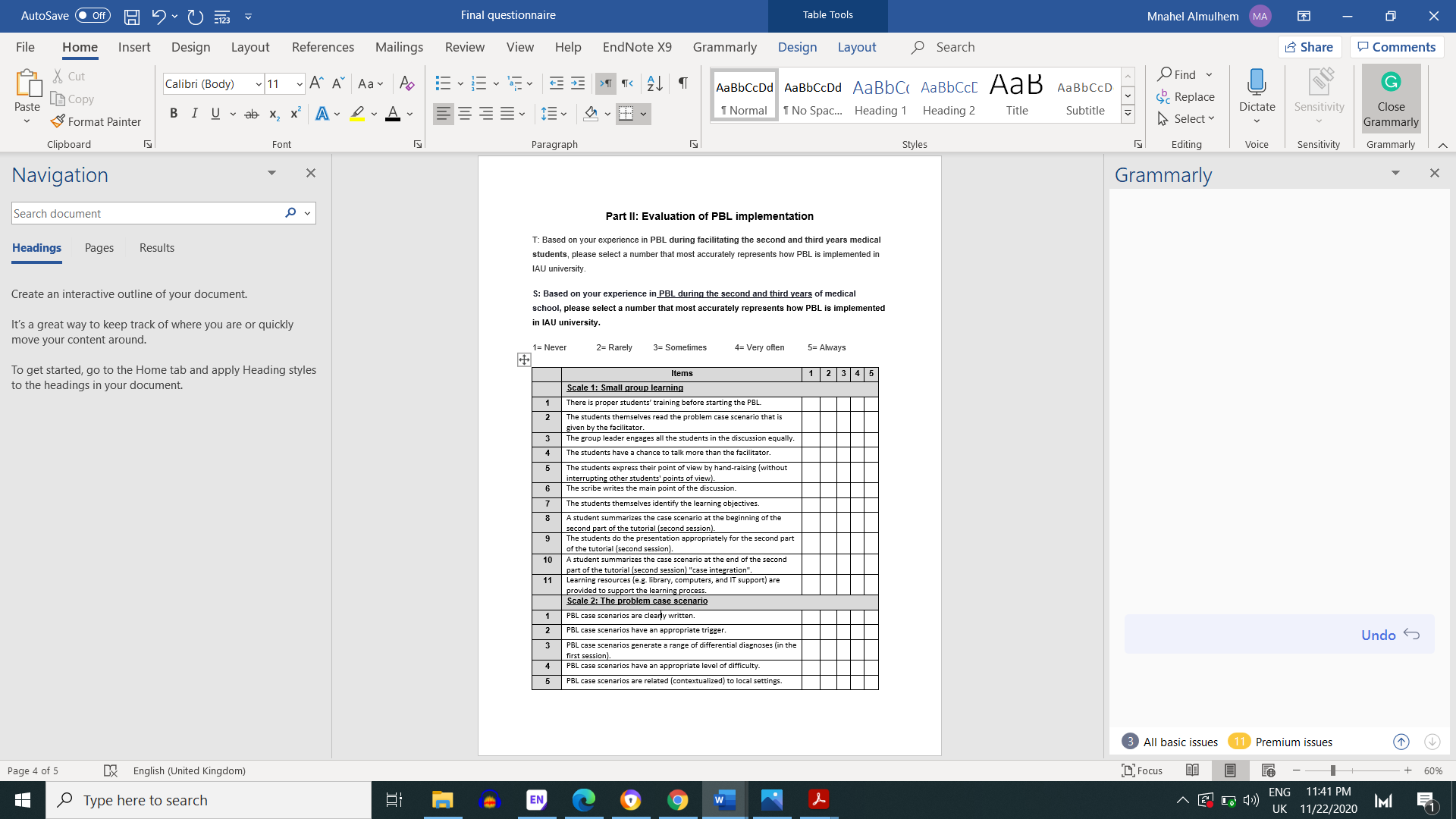


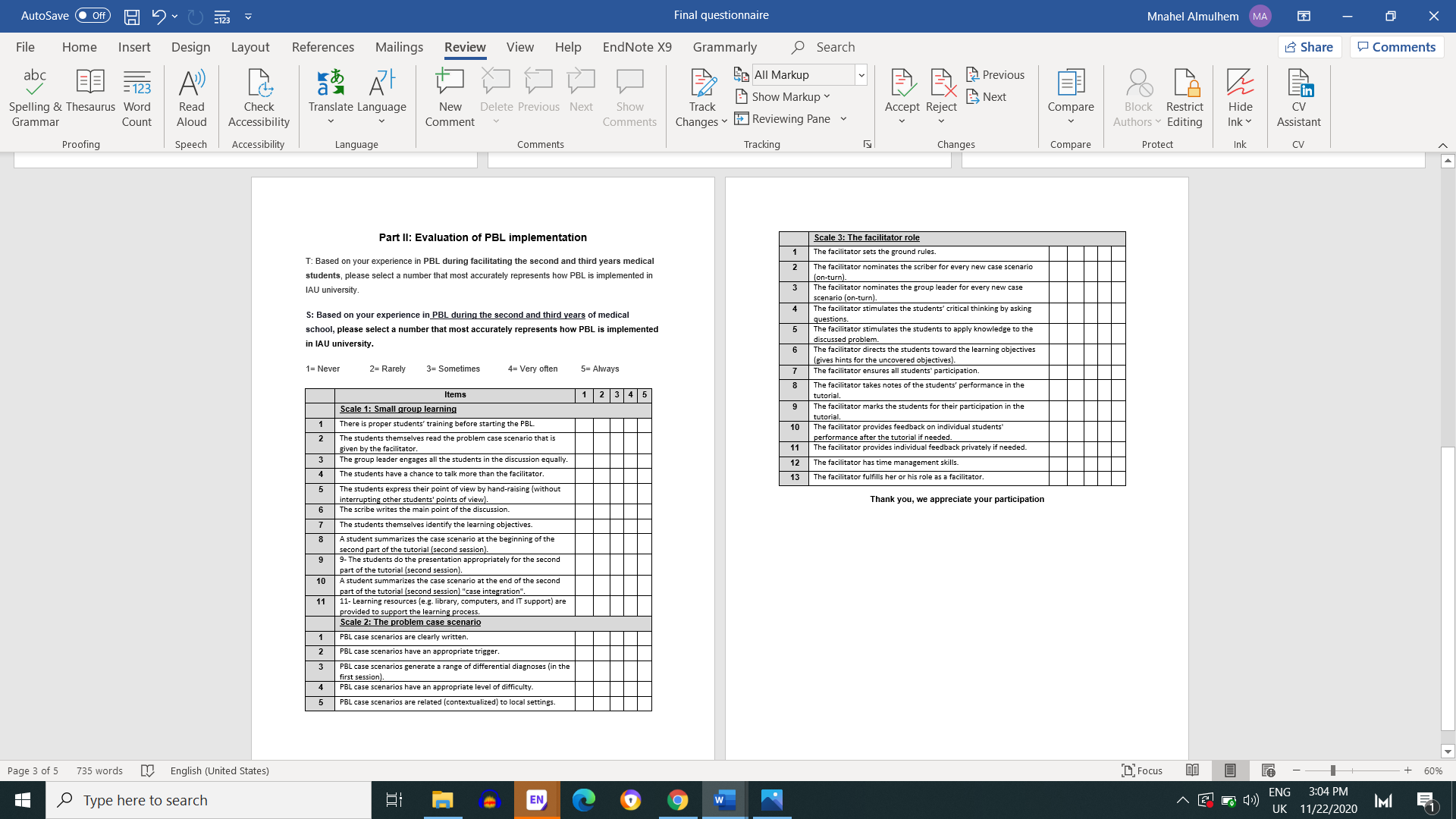


## Appendix 2: The facilitators’ characteristics and the small group learning scale

T-test for the facilitators' evaluation of the small group learning scale

| **P** | **t** | **SD** | **Mean** | **N** | **Demographics** | |
| --- | --- | --- | --- | --- | --- | --- |
| .632 | 0.482 | 4.889 | 48.42 | 24 | **Female** | **Gender** |
|  |  | 5.231 | 49.14 | 22 | **Male** |  |
| .834 | 0.211 | 5.032 | 48.73 | 44 | **Medical**  **(Graduated with MBBS)** | **Educational background** |
|  |  | 6.364 | 49.50 | 2 | **Non-Medical** |  |
| .249 | 1.167 | 5.694 | 47.74 | 19 | **Physician** | **Academic background** |
|  |  | 4.441 | 49.48 | 27 | **Academic basic scientist** |  |
| .017 | 2.485 | 3.641 | 49.79 | 34 | **Current facilitator (still a PBL facilitator)** | **Facilitation status** |
|  |  | 7.095 | 45.83 | 12 | **Previous facilitator** |  |
| .237 | 1.198 | 5.039 | 49.34 | 32 | **Yes** | **Facilitating tutorial related to the facilitators’ specialty** |
|  |  | 4.863 | 47.43 | 14 | **No** |  |
| .029 | 2.252 | 4.500 | 49.44 | 39 | **Yes** | **Attending training session before facilitation** |
|  |  | 6.377 | 45.00 | 7 | **No** |  |
| .188 | 1.338 | 4.199 | 49.21 | 38 | **Yes** | **Observing BPL tutorial before facilitation** |
|  |  | 7.873 | 46.63 | 8 | **No** |  |

ANOVA for the facilitators' evaluation of the small group learning scale

| **P** | **F** | **MS** | **df** | **SS** | **Source** | **Demographics** |
| --- | --- | --- | --- | --- | --- | --- |
| .955 | 0.108 | 2.884 | 3 | 8.651 | **Between Groups** | **Age** |
|  |  | 26.708 | 42 | 1121.718 | **Within Groups** |  |
|  |  |  | 45 | 1130.370 | **Total** |  |
| .366 | 1.129 | 27.811 | 7 | 194.674 | **Between Groups** | **Department** |
|  |  | 24.624 | 38 | 935.695 | **Within Groups** |  |
|  |  |  | 45 | 1130.370 | **Total** |  |
| .326 | 1.197 | 29.560 | 4 | 118.241 | **Between Groups** | **Academic status** |
|  |  | 24.686 | 41 | 1012.129 | **Within Groups** |  |
|  |  |  | 45 | 1130.370 | **Total** |  |
| .475 | .848 | 21.523 | 3 | 64.570 | **Between Groups** | **Number of facilitation years** |
|  |  | 25.376 | 42 | 1065.800 | **Within Groups** |  |
|  |  |  | 45 | 1130.370 | **Total** |  |
| .357 | 1.055 | 26.430 | 2 | 52.859 | **Between Groups** | **Academic year of facilitation** |
|  |  | 25.058 | 43 | 1077.510 | **Within Groups** |  |
|  |  |  | 45 | 1130.370 | **Total** |  |

## Appendix 3: The facilitators’ characteristics and the problem case scenario scale

T-test for the facilitators' evaluation of the problem case scenario scale

| **P** | **t** | **SD** | **Mean** | **N** | **Demographics** | |
| --- | --- | --- | --- | --- | --- | --- |
| .161 | 1.424 | 2.667 | 21.38 | 24 | **Female** | **Gender** |
|  |  | 2.686 | 22.50 | 22 | **Male** |  |
| .631 | .484 | 2.728 | 21.95 | 44 | **Medical**  **(Graduated with MBBS)** | **Educational background** |
|  |  | 2.828 | 21.00 | 2 | **Non-Medical** |  |
| .090 | 1.736 | 3.178 | 21.11 | 19 | **Physician** | **Academic background** |
|  |  | 2.208 | 22.48 | 27 | **Academic basic scientist** |  |
| .017 | 2.483 | 2.149 | 22.47 | 34 | **Current facilitator (still a PBL facilitator)** | **Facilitation status** |
|  |  | 3.525 | 20.33 | 12 | **Previous facilitator** |  |
| .004 | 3.067 | 2.548 | 22.66 | 32 | **Yes** | **Facilitating tutorial related to the facilitators’ specialty** |
|  |  | 2.326 | 20.21 | 14 | **No** |  |
| .084 | 1.768 | 2.557 | 22.21 | 39 | **Yes** | **Attending training session before facilitation** |
|  |  | 3.147 | 20.29 | 7 | **No** |  |
| .452 | .759 | 2.503 | 22.05 | 38 | **Yes** | **Observing BPL tutorial before facilitation** |
|  |  | 3.655 | 21.25 | 8 | **No** |  |

ANOVA for the facilitators' evaluation of the problem case scenario scale

| **P** | **F** | **MS** | **Df** | **SS** | **Source** | **Demographics** |
| --- | --- | --- | --- | --- | --- | --- |
| .722 | 0.446 | 3.391 | 3 | 10.174 | **Between Groups** | **Age** |
|  |  | 7.607 | 42 | 319.478 | **Within Groups** |  |
|  |  |  | 45 | 329.652 | **Total** |  |
| .515 | 0.902 | 6.713 | 7 | 46.988 | **Between Groups** | **Department** |
|  |  | 7.439 | 38 | 282.664 | **Within Groups** |  |
|  |  |  | 45 | 329.652 | **Total** |  |
| .465 | 0.913 | 6.742 | 4 | 26.967 | **Between Groups** | **Academic status** |
|  |  | 7.383 | 41 | 302.685 | **Within Groups** |  |
|  |  |  | 45 | 329.652 | **Total** |  |
| .379 | 1.053 | 7.684 | 3 | 23.052 | **Between Groups** | **Number of facilitation years** |
|  |  | 7.300 | 42 | 306.600 | **Within Groups** |  |
|  |  |  | 45 | 329.652 | **Total** |  |
| .945 | .057 | .433 | 2 | .865 | **Between Groups** | **Academic year of facilitation** |
|  |  | 7.646 | 43 | 328.787 | **Within Groups** |  |
|  |  |  | 45 | 329.652 | **Total** |  |

## Appendix 4: The facilitators’ characteristics and the facilitator role scale

T-test for the facilitators' evaluation of the facilitator role scale

| **P** | **t** | **SD** | **Mean** | **N** | **Demographics** | |
| --- | --- | --- | --- | --- | --- | --- |
| .909 | 0.116 | 5.790 | 59.29 | 24 | **Female** | **Gender** |
|  |  | 8.510 | 59.05 | 22 | **Male** |  |
| .716 | 0.366 | 7.239 | 59.09 | 44 | **Medical**  **(Graduated with MBBS)** | **Educational background** |
|  |  | 5.657 | 61.00 | 2 | **Non-Medical** |  |
| .313 | 1.020 | 9.140 | 57.89 | 19 | **Physician** | **Academic background** |
|  |  | 5.320 | 60.07 | 27 | **Academic basic scientist** |  |
| .224 | 1.234 | 5.438 | 59.94 | 34 | **Current facilitator (still a PBL facilitator)** | **Facilitation status** |
|  |  | 10.617 | 57.00 | 12 | **Previous facilitator** |  |
| .440 | .779 | 7.726 | 59.72 | 32 | **Yes** | **Facilitating tutorial related to the facilitators’ specialty** |
|  |  | 5.622 | 57.93 | 14 | **No** |  |
| .875 | .158 | 7.297 | 59.10 | 39 | **Yes** | **Attending training session before facilitation** |
|  |  | 6.680 | 59.57 | 7 | **No** |  |
| .152 | 1.456 | 5.194 | 59.87 | 38 | **Yes** | **Observing BPL tutorial before facilitation** |
|  |  | 13.032 | 55.88 | 8 | **No** |  |

ANOVA for the facilitators' evaluation of the facilitator role scale

| **P** | **F** | **MS** | **df** | **SS** | **Source** | **Demographics** |
| --- | --- | --- | --- | --- | --- | --- |
| .748 | 0.409 | 21.673 | 3 | 65.019 | **Between Groups** | **Age** |
|  |  | 53.038 | 42 | 2227.590 | **Within Groups** |  |
|  |  |  | 45 | 2292.609 | **Total** |  |
| .169 | 1.586 | 74.044 | 7 | 518.311 | **Between Groups** | **Department** |
|  |  | 46.692 | 38 | 1774.298 | **Within Groups** |  |
|  |  |  | 45 | 2292.609 | **Total** |  |
| .352 | 1.138 | 57.288 | 4 | 229.152 | **Between Groups** | **Academic status** |
|  |  | 50.328 | 41 | 2063.456 | **Within Groups** |  |
|  |  |  | 45 | 2292.609 | **Total** |  |
| .388 | 1.033 | 52.492 | 3 | 157.475 | **Between Groups** | **Number of facilitation years** |
|  |  | 50.837 | 42 | 2135.133 | **Within Groups** |  |
|  |  |  | 45 | 2292.609 | **Total** |  |
| .032 | 3.727 | 169.373 | 2 | 338.745 | **Between Groups** | **Academic year of facilitation** |
|  |  | 45.439 | 43 | 1953.864 | **Within Groups** |  |
|  |  |  | 45 | 2292.609 | **Total** |  |

Multiple comparisons result by type of academic year of facilitation (Tukey post-hoc test)

| **type of Academic year of facilitation** | **N** | **Mean** | **Second year** | **Third year** | **Both second and third years** |
| --- | --- | --- | --- | --- | --- |
| **Second year** | 22 | 60.23 | -- | .080 | .758 |
| **Third year** | 13 | 55.00 | .080 | -- | .039* |
| **Both second and third years** | 11 | 62.00 | .758 | .039* | -- |

## Appendix 5: The students’ characteristics and the small group learning scale

T-test for the student’s evaluation of the small group learning scale

| **P** | **t** | **SD** | **Mean** | **N** | **Demographics** | |
| --- | --- | --- | --- | --- | --- | --- |
| .000 | 7.513 | 5.354 | 42.56 | 221 | **Female** | **Gender** |
|  |  | 7.796 | 36.97 | 103 | **Male** |  |

ANOVA for the students’ evaluation of the small group learning scale

| **P** | **F** | **MS** | **df** | **SS** | **Source** | **Demographics** |
| --- | --- | --- | --- | --- | --- | --- |
| .000 | 10.183 | 416.028 | 4 | 1664.110 | **Between Groups** | **Age** |
|  |  | 40.857 | 319 | 13033.331 | **Within Groups** |  |
|  |  |  | 323 | 14697.441 | **Total** |  |
| .000 | 8.335 | 347.672 | 4 | 1390.688 | **Between Groups** | **Academic year the student has completed** |
|  |  | 41.714 | 319 | 13306.753 | **Within Groups** |  |
|  |  |  | 323 | 14697.441 | **Total** |  |

Multiple comparisons table for the student’s age categories (Tukey post-hoc test)

| **Type of students age** | **N** | **Mean** | **18-19** | **20-21** | **22-23** | **24-25** | **more than 26** |
| --- | --- | --- | --- | --- | --- | --- | --- |
| **18-19** | 27 | 44.81 | -- | .243 | .015* | .000* | .190 |
| **20-21** | 119 | 42.02 | .243 | -- | .367 | .000* | .550 |
| **22-23** | 129 | 40.54 | .015* | .367 | -- | .001* | .792 |
| **24-25** | 46 | 36.17 | .000* | .000* | .001* | -- | 1.000 |
| **more than 26** | 3 | 36.33 | .190 | .550 | .792 | 1.000 | -- |

Multiple comparisons table for the student’s academic year they have completed (Tukey post-hoc test)

| **Type of Academic year the students have completed** | **N** | **Mean** | **Second-year** | **Third-year** | **Fourth-year** | **Fifth-year** | **Sixth- year** |
| --- | --- | --- | --- | --- | --- | --- | --- |
| **Second year** | 62 | 43.44 | -- | .554 | .527 | .031* | .000* |
| **Third year** | 52 | 41.60 | .554 | -- | 1.000 | .753 | .003* |
| **Fourth year** | 72 | 41.69 | .527 | 1.000 | -- | .627 | .001* |
| **Fifth year** | 75 | 40.20 | .031* | .753 | .627 | -- | .047* |
| **Sixth year** | 63 | 37.14 | .000* | .003* | .001* | .047* | -- |

## Appendix 6: The students’ characteristics and the problem case scenario scale

T-test for the student’s evaluation of the problem case scenario scale

| **P** | **t** | **SD** | **Mean** | **N** | **Demographics** | |
| --- | --- | --- | --- | --- | --- | --- |
| .000 | 4.074 | 3.407 | 20.81 | 221 | **Female** | **Gender** |
|  |  | 4.542 | 18.96 | 103 | **Male** |  |

ANOVA for the students’ evaluation of the problem case scenario scale

| **P** | **F** | **MS** | **Df** | **SS** | **Source** | **Demographics** |
| --- | --- | --- | --- | --- | --- | --- |
| .009 | 3.449 | 50.766 | 4 | 203.063 | **Between Groups** | **Age** |
|  |  | 14.718 | 319 | 4694.937 | **Within Groups** |  |
|  |  |  | 323 | 4898.000 | **Total** |  |
| .041 | 2.521 | 37.526 | 4 | 150.103 | **Between Groups** | **Academic year the student completed** |
|  |  | 14.884 | 319 | 4747.897 | **Within Groups** |  |
|  |  |  | 323 | 4898.000 | **Total** |  |

Multiple comparisons table for the student’s age categories (Tukey post-hoc test)

| **Type of students age** | **N** | **Mean** | **18-19** | **20-21** | **22-23** | **24-25** | **more than 26** |
| --- | --- | --- | --- | --- | --- | --- | --- |
| **18-19** | 27 | 21.33 | -- | .893 | .721 | .023* | .517 |
| **20-21** | 119 | 20.59 | .893 | -- | .981 | .018* | .690 |
| **22-23** | 129 | 20.32 | .721 | .981 | -- | .052 | .761 |
| **24-25** | 46 | 18.52 | .023* | .018* | .052 | -- | .996 |
| **more than 26** | 3 | 17.67 | .517 | .690 | .761 | .996 | -- |

Multiple comparisons table for the student’s academic year they have completed (Tukey post-hoc test)

| **Type of Academic year the students have completed** | **N** | **Mean** | **Second-year** | **Third-year** | **Fourth-year** | **Fifth-year** | **Sixth- year** |
| --- | --- | --- | --- | --- | --- | --- | --- |
| **Second-year** | 62 | 20.79 | -- | .956 | .985 | .136 | .015* |
| **Third year** | 52 | 20.75 | .956 | -- | .968 | .173 | .023* |
| **Fourth year** | 72 | 20.78 | .985 | .968 | -- | .126 | .012* |
| **Fifth year** | 75 | 19.80 | .136 | .173 | .126 | -- | .286 |
| **Sixth year** | 63 | 19.10 | .015* | .023* | .012* | .286 | -- |

## Appendix 7: The students’ characteristics and the facilitator role scale

T-test for the student’s evaluation of the facilitator role scale

| **P** | **t** | **SD** | **Mean** | **N** | **Demographics** | |
| --- | --- | --- | --- | --- | --- | --- |
| .000 | 5.096 | 7.526 | 53.42 | 221 | **Female** | **Gender** |
|  |  | 11.049 | 48.07 | 103 | **Male** |  |

ANOVA for the students’ evaluation of the facilitator role scale

| **P** | **F** | **MS** | **Df** | **SS** | **Source** | **Demographics** |
| --- | --- | --- | --- | --- | --- | --- |
| .000 | 7.093 | 549.726 | 4 | 2198.905 | **Between Groups** | **Age** |
|  |  | 77.508 | 319 | 24724.971 | **Within Groups** |  |
|  |  |  | 323 | 26923.877 | **Total** |  |
| .001 | 4.652 | 371.013 | 4 | 1484.052 | **Between Groups** | **Academic year the student completed** |
|  |  | 79.749 | 319 | 25439.825 | **Within Groups** |  |
|  |  |  | 323 | 26923.877 | **Total** |  |

Multiple comparisons table for the student’s age categories (Tukey post-hoc test)

| **Type of students age** | **N** | **Mean** | **18-19** | **20-21** | **22-23** | **24-25** | **more than 26** |
| --- | --- | --- | --- | --- | --- | --- | --- |
| **18-19** | 27 | 56.93 | -- | .132 | .046 | .000* | .996 |
| **20-21** | 119 | 52.51 | .132 | -- | .961 | .001* | .989 |
| **22-23** | 129 | 51.75 | .046 | .961 | -- | .003* | .970 |
| **24-25** | 46 | 46.28 | .000* | .001* | .003* | -- | .459 |
| **more than 26** | 3 | 55.00 | .996 | .989 | .970 | .459 | -- |

Multiple comparisons table for the student’s academic year they have completed (Tukey post-hoc test)

| **Type of Academic year the students have completed** | **N** | **Mean** | **Second-year** | **Third-year** | **Fourth-year** | **Fifth-year** | **Sixth- year** |
| --- | --- | --- | --- | --- | --- | --- | --- |
| **Second year** | 62 | 54.77 | -- | .517 | .756 | .043 | .001* |
| **Third year** | 52 | 52.13 | .517 | -- | .988 | .843 | .180 |
| **Fourth year** | 72 | 52.93 | .756 | .988 | -- | .458 | .031* |
| **Fifth year** | 75 | 50.48 | .043* | .843 | .458 | -- | .670 |
| **Sixth year** | 63 | 48.44 | .001* | .180 | .031* | .670 | -- |

## Appendix 8: Comparison between the facilitators’ and students’ evaluations

T-test to compare the facilitators' and students' evaluations of the evaluation scales

| **P** | **T** | **Std. Deviation** | **Mean** | **N** | **Participant** | **Scale** |
| --- | --- | --- | --- | --- | --- | --- |
| .000 | 7.723 | 5.012 | 48.76 | 46 | **Facilitators** | **Small group learning** |
|  |  | 6.746 | 40.78 | 324 | **Students** |  |
| .005 | 2.847 | 2.707 | 21.91 | 46 | **Facilitators** | **Problem case scenario** |
|  |  | 3.894 | 20.22 | 324 | **Students** |  |
| .000 | 5.312 | 7.138 | 59.17 | 46 | **Facilitators** | **Facilitator role** |
|  |  | 9.130 | 51.72 | 324 | **Students** |  |
